# A robust and reproducible human pluripotent stem cell derived model of neurite outgrowth in a three-dimensional culture system and its application to study neurite inhibition

**DOI:** 10.1016/j.neuint.2016.12.009

**Published:** 2017-06

**Authors:** Kirsty E. Clarke, Daniel M. Tams, Andrew P. Henderson, Mathilde F. Roger, Andrew Whiting, Stefan A. Przyborski

**Affiliations:** a Department of Biosciences, Durham University, South Road, Durham DH1 3LE, UK; b Department of Chemistry, Durham University, South Road, Durham DH1 3LE, UK; c ReproCELL Europe Ltd., NETPark Incubator, Thomas Wright Way, Sedgefield TS21 3FD, UK

**Keywords:** Stem cell, Neuronal, Neuritogenesis, 3D culture, Cell-differentiation, Retinoid acid

## Abstract

The inability of neurites to grow and restore neural connections is common to many neurological disorders, including trauma to the central nervous system and neurodegenerative diseases. Therefore, there is need for a robust and reproducible model of neurite outgrowth, to provide a tool to study the molecular mechanisms that underpin the process of neurite inhibition and to screen molecules that may be able to overcome such inhibition. In this study a novel in vitro pluripotent stem cell based model of human neuritogenesis was developed. This was achieved by incorporating additional technologies, notably a stable synthetic inducer of neural differentiation, and the application of three-dimensional (3D) cell culture techniques. We have evaluated the use of photostable, synthetic retinoid molecules to promote neural differentiation and found that 0.01 μM EC23 was the optimal concentration to promote differentiation and neurite outgrowth from human pluripotent stem cells within our model. We have also developed a methodology to enable quick and accurate quantification of neurite outgrowth derived from such a model. Furthermore, we have obtained significant neurite outgrowth within a 3D culture system enhancing the level of neuritogenesis observed and providing a more physiological microenvironment to investigate the molecular mechanisms that underpin neurite outgrowth and inhibition within the nervous system. We have demonstrated a potential application of our model in co-culture with glioma cells, to recapitulate aspects of the process of neurite inhibition that may also occur in the injured spinal cord. We propose that such a system that can be utilised to investigate the molecular mechanisms that underpin neurite inhibition mediated via glial and neuron interactions.

## Highlights

• Development of a robust, novel neurite outgrowth assay from human pluripotent stem cell derived neural cell aggregates.
• Synthetic retinoids induce neural differentiation of pluripotent stem cells to a greater extent than natural ATRA.
• Neurospheres cultured on a 3D scaffold provide a more physiologically relevant model of neurite outgrowth.
• Suppression of neurite outgrowth by glioma cells in 3D enables the study of neurite inhibitory mechanisms in the glial scar.

## Graphical abstract

**Figure:**
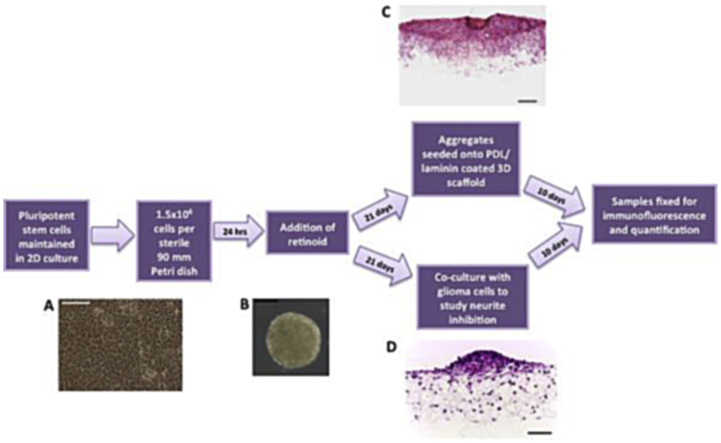

## 1 Introduction

During neuronal development, dynamic processes involving cytoskeletal remodelling occur, known as neuritogenesis. Long cytoskeletal processes, known as neurites, project from the cell body of the developing neuron and ultimately form distant neural connections that become the axon and dendrites of a mature neural network (Da Silva and Dotti, 2002). The growth cone is situated at the distal tip of the developing neurite and is rich in actin filaments (Khodosevich and Monyer, 2010) and microtubules (Dent et al., 2011), along with adaptor proteins, as it undergoes significant cytoskeletal rearrangements during neurite elongation. Primarily the remodelling of the actin cytoskeleton drives protrusion and motility of the growth cone; therefore actin dynamics play an important role during neuritogenesis (Dent et al., 2011).

Complex neuronal networks are essential for nervous system function, and depend on the successful formation of neurite projections from the developing neuron. Accordingly, neuritogenesis is an integral process essential to the proper functioning of the nervous system (Da Silva and Dotti, 2002). Inhibition of neurite outgrowth is common to many nervous system disorders including central nervous system trauma (Rolls et al., 2009, Yiu and He, 2006a, Niederost et al., 2002, Bush et al., 1999) and neurodegenerative diseases (Petratos et al., 2008, Postuma et al., 2000, Takenouchi et al., 2001, Winner et al., 2011). This highlights the importance of *in vitro* models of neuritogenesis to enhance our understanding of the process and to screen potential therapeutic molecules.

Many of the popular current models of neurite outgrowth include the isolation and *in vitro* culture of primary mammalian cell types derived from the nervous system of rats and mice. Dorsal root ganglia (DRG) neurons and explants derived from mammalian or chick embryos are commonly isolated and cultured *in vitro* without the need for differentiation and form the basis of many neurite outgrowth studies (Clagett-Dame et al., 2006, Balgude et al., 2001, Fitzgerald et al., 1993). Although the use of primary cells avoids the limitations associated with immortalised cell lines, the physiology of animal derived cells may differ significantly from that of human cells and impact experimental outcomes. This becomes particularly important when applying such models to human neurological diseases. Human cell lines such as SH-SY5Y are commonly used to study neurite outgrowth (Ferrari-Toninelli et al., 2004, Tucholski et al., 2001, Ross et al., 1983). SH-SY5Y cells are a popular neuroblastoma cell line that originated from a metastatic bone biopsy that require differentiation with *all-*trans retinoic acid (ATRA) (Kovalevich and Langford, 2013). However, due to their neoplastic nature, mixed morphology, and somewhat limited capacity for neuritogenesis, questions have arisen as to the consistency and appropriateness of the use of such cells in neurite outgrowth models, particularly when the application may be to test potential therapeutics (Kovalevich and Langford, 2013).

Human pluripotent stem cells are recognised models of neural development. The embryonal carcinoma (EC) stem cell line, TERA2, readily differentiates into neuronal subtypes when exposed to ATRA. EC cells are the malignant counterpart of embryonic stem cells, and cells from the TERA2 lineage have provided the basis for many *in vitro* models of neural differentiation, function and neurite outgrowth (Tegengem et al., 2011, Satoh et al., 1997, Pewsey et al., 2010, Roloff et al., 2015, Przyborski, 2001, Przyborski et al., 2000, Przyborski et al., 2003, Przyborski et al., 2004, Stewart et al., 2004, Coyne et al., 2011). While such models are valuable, there is significant scope to enhance their reliability in terms of robustness and reproducibility.

Cell technologies are becoming available which are designed to improve current practice, and enhance the development and application of *in vitro* assays. Such techniques can be used to improve the robustness and reproducibility of neurite outgrowth assays and enhance their physiological significance. For example, ATRA is a derivative of vitamin A, which is important during the patterning and differentiation of the developing nervous system *in vivo* (Magden, 2007). However, the use of ATRA *in vitro* is limited, as it readily breaks down when exposed to light and heat. Stable, synthetic forms of retinoic acid such as, EC23 and AH61 offer more practical *in vitro* use, as they contain a non-isomerisable conjugated linker unit that stabilises the molecule, and have previously been described as tools for stem cell differentiation studies (Clemens et al., 2013, Christie et al., 2008). In recent years there has been growing interest in 3D culture techniques to enhance the physiological relevance of *in vitro* models. Many existing models of neurite outgrowth use conventional two-dimensional (2D) cell culture, rather than culturing developing neurites in a more physiological three-dimensional (3D) system. 3D scaffolds have been developed to enhance neurite development (Hayman et al., 2004, Hayman et al., 2005). Another limitation of current 2D neurite outgrowth models is that they are difficult to quantify, as monolayers of developing neurons intertwine and form complex neuronal networks within the cell population, making it difficult to identify individual neurites emanating from individual perikarya. We propose that quantification of neurite outgrow can be simplified using a neurosphere model, whereby all neurites radiate from a central point aiding quantification and significantly reducing neurite network complexity.

In this study we have combined our stem cell model of human neuritogenesis with synthetic retinoids and 3D cell culture technologies to produce an enhanced model of neurite development and outgrowth. This provides a powerful new tool to study neurite inhibition and to investigate the molecular processes involved in the context of different neurological disorders. Here, we present an example application of this system, to study the process of neurite inhibition and the ability of small molecules to overcome such inhibition. Such research tools will be important to help elucidate the mechanisms that underpin neurite inhibition to enable intervention and recovery of neurite outgrowth.

## 2 Material & methods

### 2.1 Cell line maintenance

#### 2.1.1 Human pluripotent stem cells

The EC cell line TERA2.cl.SP12, was maintained in maintenance medium consisting of Dulbecco's modified Eagles medium containing high glucose (DMEM-HG, Lonza, Basel, Switzerland), 10% heat treated foetal bovine serum (FBS, ThermoFisher Scientific, Cramlington, UK), 2 mM l-glutamine (Lonza) and 20 active units of penicillin and streptomycin (Lonza). EC cells were maintained at high confluence and passaged using acid washed glass beads in a 2–3 ratio into 75 cm (Khodosevich and Monyer, 2010) culture flasks (BD Falcon, Erembodegem, Belgium) to ensure their pluripotent phenotype. A Leica DFC 310FX with digital camera DMI 3000B was used to record phase contrast images of live cells.

#### 2.1.2 U-118 MG glioma cells

U-118 MG cells were maintained in DMEM-HG, 10% FBS, 2 mM l-glutamine and 100 active units of penicillin and streptomycin. Cells were maintained in 75 cm^2^ culture flasks (ThermoFisher Scientific) and passaged at high confluence in a 3–6 ratio using 0.25% trypsin/2 mM EDTA (Lonza).

### 2.2 Induction of cell differentiation in conventional 2D culture

TERA2.cl.SP12 stem cells were seeded at 250,000 cells per 25 cm (Khodosevich and Monyer, 2010) culture flask (BD Falcon) and incubated for 24 h to allow cells to adhere. Following 24 h incubation, the culture medium was replaced with media containing ATRA Sigma-Aldrich, EC23 (ReproCELL Europe, UK) or AH61 (Chemistry Department, Durham, UK) in dimethyl sulfoxide (DMSO) at concentrations of 0.001 μM, 0.01 μM, or 1 μM. Cells were cultured for a further 7 days with a media change on the 4th day prior to analysis of cell surface markers by flow cytometry or lysis for Q-PCR.

### 2.3 Analysis of protein expression by flow cytometry

Following the 7 day differentiation period a single cell suspension was obtained through the trypsinisation of monolayers and cells were resuspended in blocking buffer (0.1% bovine serum albumin (BSA, Sigma-Aldrich) in phosphate buffered saline (PBS)) at a density of 0.2 × 10^6^ cells per well of a 96 well plate (Greiner Bio-one, Stonehouse, UK). The plate was centrifuged at 1000 rpm for 3 min at 4 °C, and the pellet was resuspended in the appropriate primary antibody (anti-p3X (gift from Prof. Peter Andrews, Sheffield University, UK), anti-SSEA3 (Developmental Studies Hybridoma Bank, Iowa, USA), anti-TRA-160 (Millipore, Darmstadt, Germany) or anti-A2B5 (R&D Systems, Abingdon, UK)) for 60 min. The cells were then washed 3 times in blocking buffer prior to incubation with the secondary antibody (anti-mouse IgM (Sigma-Aldrich)) for 60 min. Cells were then washed a further three times in blocking buffer and resuspended in blocking buffer prior to analysis using the Guava Easy Cyte Plus and Cytosoft software (Millipore), and settings adjusted to the negative control (P3X), a murine marker that is not be expressed on human cells to take non-specific staining into account.

### 2.4 Real time quantitative polymerase chain reaction (Q-PCR)

Q-PCR was carried out on cells immediately following lysis. Commercial RNA extraction (Qiagen) and reverse transcription (ThermoFisher) kits were used following manufacturer's instructions. Following lysis, cells were homogenised using a 20-gauge needle and passed through an RNeasy spin column. DNase digestion was carried out prior to RNA extraction from the column as a suspension in RNase free water. RNA quantity was determined using a Nanodrop Spectrophotometer ND-100™, followed by analysis on a 1% agarose gel. Reverse transcription was carried out using a high-capacity cDNA reverse transcription kit and a Biometra T1 thermal cycler. The expression of genes (*Nanog* (ThermoFisher), *Oct-4* (ThermoFisher) and *Pax6* (ThermoFisher)) was quantified against *Glyceraldehyde-3-phosphate dehydrogenase* (*GAPDH*, ThermoFisher) and Q-PCR was analysed using an Applied Biosystems RT-PCR system.

### 2.5 Formation of neurospheres

To form stem cell aggregates, cells were trypsinised and seeded in a single cell suspension at a density of 1.5 × 10^6^ cells per sterile, on an untreated 90 mm Petri dish (ThermoFisher Scientific) and incubated for 24 h prior to retinoid treatment. Following 24 h, retinoid compounds were added directly to induce neural differentiation; cultures were further maintained with retinoid compounds for 21 days with media changes, replenishing retinoid treatment, twice per week.

### 2.6 Induction of neurite outgrowth

Following 21 days, differentiated aggregates, were seeded into either 48-well culture plates or 12-well format Alvetex^®^ scaffold inserts (ReproCELL Europe, UK). Alvetex^®^ scaffolds were rendered hydrophilic by plasma treatment using the K1050× Plasma Asher at a power level of 40 W, for 5 min. Treated scaffolds and conventional culture plates were coated overnight at room temperature with 10 μgml^−1^ poly-d-lysine (Sigma-Aldrich) and 10 μgml^−1^ laminin (Sigma-Aldrich) solution. Stem cell aggregates were subsequently maintained for a further 10 days with mitotic inhibitors: 1 μM cytosine arabinoside (Sigma-Aldrich), 10 μM 5'fluoro2'deoxyuridine (Sigma-Aldrich) and 10 μM uridine (Sigma-Aldrich) on coated Alvetex^®^ or culture plates. Cells were then fixed with 4% paraformaldehyde (PFA) in PBS for 60 min and processed for immunofluorescence and histological analysis.

### 2.7 Co-culture of glioma cells and neurospheres

Glioma cells (U-118 MG), were seeded at a density of 1 × 10^6^ cells in a volume of 100 μL of media to the surface of a prepared scaffold and allowed to settle for 15 min at 37 °C. Neurospheres were then added to the surface of the scaffold as previously described in Section 2.6 and incubated in the presence or absence of 10 μM Y-27632 (a selective p160 ROCK inhibitor) and mitotic inhibitors for 10 days, and subsequently fixed with 4% PFA for processing and analysis.

### 2.8 Immunofluorescent analysis of 2D cultures and 3D models

Both 2D and 3D cultures fixed with 4% PFA were permeabilised with 0.1% Triton X-100 (Sigma-Aldrich) in PBS for 10 min and blocked on ice for 60 min with 1% goat serum (Sigma-Aldrich) and 0.01% Tween (Sigma-Aldrich) in PBS. 2D cell culture samples were then incubated with primary antibodies (anti-β—III—tubulin (Cambridge bioscience, Cambridge, UK), anti-nestin (Abcam, Cambridge, UK), anti-Neurofilament-H (Abcam), anti-neurofilament-M (Sigma-Aldrich) or anti-neurofilment-L (Sigma-Aldrich)) on ice for 60 min, whereas cells on Alvetex^®^ scaffolds were incubated for 120 min before being washed 3 times in blocking buffer. Samples were then incubated with the relevant secondary antibody Goat anti-rabbit Alexa Fluor^®^ 488 (ThermoFisher Scientific) or Goat anti-mouse Alexa Fluor^®^ 488 (ThermoFisher Scientific) along with the nuclear dye Hoechst 33,342 (ThermoFisher Scientific) for a further 60 min. Samples were then washed 3 times in blocking buffer and 2D samples were stored in PBS until microscopy, whereas cells on Alvetex^®^ scaffolds were whole mounted onto microscope slides (ThermoFisher Scientific) using Vectashield (Vecta Laboratories, California, USA) fluorescence mounting media. Samples were imaged using either the Zeiss 880 and Zeiss 510 confocal microscopes with Zen software, or the Leica SP5 confocal microscope with Leica Application Suite software.

### 2.9 Paraffin embedding of 3D models

Fixed 3D cultures were dehydrated in 30% followed by 50% ethanol. Samples were stained in 0.1% (w/v) crystal violet (Sigma-Aldrich) dissolved in 70% ethanol to visualise the neurospheres and further dehydrated through a series of ethanol baths. Samples were then bathed in histoclear (ThermoFisher Scientific) for 15 min followed by 50% histoclear, 50% paraffin wax mix (ThermoFisher Scientific) for 30 min at 60 °C. Samples were further incubated for 60 min with melted paraffin wax only at 60 °C before being embedded using plastic moulds (CellPath, Newton, UK) and embedding cassettes (ThermoFisher Scientific) to allow for transverse sectioning.

### 2.10 Haematoxylin and eosin staining of paraffin embedded sections

Alvetex^®^ Scaffold 3D paraffin embedded samples were sectioned at 6 μm using a Leica Microtome RM2125RT and mounted onto charged superfrost microscope slides (ThermoFisher Scientific). Sections were deparaffinised in Histoclear and rehydrated. Slides were incubated in Mayer's haematoxylin (Sigma-Aldrich) for 5 min and rinsed in distilled water for 30 s before being incubated with alkaline alcohol for 30 s to ensure the nuclei appear blue. Samples were then dehydrated, before being incubated with eosin (Sigma-Aldrich) for 1 min and then further dehydrated and incubated in Histoclear for 3 min prior to mounting with DPX (ThermoFisher Scientific) ready for microscopy using a Leica ICC50 high definition camera mounted onto a Leica microscope.

### 2.11 Immunofluorescent analysis of 3D cultures

Alvetex^®^ Scaffold 3D samples were deparaffinised and rehydrated as previously described. Antigen retrieval was achieved by incubating samples in a 95 °C water bath for 20 min with citrate buffer anhydrous citric acid (Sigma-Aldrich, dissolved in (DI) water). Samples were then permeabilised with 0.1% Triton X-100 in PBS for 20 min and blocked with 1% goat serum (Sigma-Aldrich) and 0.01% Tween in PBS for 30 min. Primary antibody (anti-β—III—tubulin, anti-chondroitin sulphate (Abcam), or anti-brevican (Santa Cruz Biotechnology, Heidelberg, Germany)) was incubated with the samples overnight at 4 °C, followed by three washes in blocking buffer. Samples were then incubated with the relevant secondary antibody (Goat anti-rabbit Alexa Fluor^®^ 488 or Goat anti-mouse Alexa Fluor^®^ 488), along with the nuclear dye Hoechst 33,342 for a further 60 min before being washed 3 times for 15 min in blocking buffer and mounted using Vectashield fluorescence mounting media. Confocal images were obtained using the Zeiss 880 and Zeiss 510 confocal microscopes with Zen software, or the Leica SP5 confocal microscope with Leica Application Suite software.

### 2.12 Image analysis

Image J software was used to quantify neurite outgrowth, through calibrating the scale of the software and tracing individual neurites. A sampling method was employed to count neurites per neurosphere when grown in 2D culture, whereby each image was overlaid with a grid and a random number generator (random.org) used to select 3 squares per image to quantify. Neurites from neurospheres differentiated with ATRA and cultured in 2D, were quantified using both the sampling method and tracing every neurite per aggregate and the outcomes of both methods were subsequently compared. A two-way ANOVA was used to assess the statistical significance of data generated. Our experiments showed that for each parameter measured (number and density of neurites), there was no significant difference between counting all neurites or using the sampling approach. Therefore the sampling method was selected and applied to the remainder of the study. In 3D cultures using Alvetex^®^ Scaffold, a sampling method was not used and all neurites that visibly penetrated the scaffold were counted.

### 2.13 Statistical analysis

GraphPad Prism was used to assess the statistical significance of results and in all cases the statistical test ANOVA was conducted to determine significance with * = p ≤ 0.05, ** = p ≤ 0.01, *** = p ≤ 0.001, **** = p ≤ 0.0001.

## 3 Results

### 3.1 Application of synthetic molecules to promote robust neural differentiation of 2D monolayers of human pluripotent stem cells

**Fig. 1:**
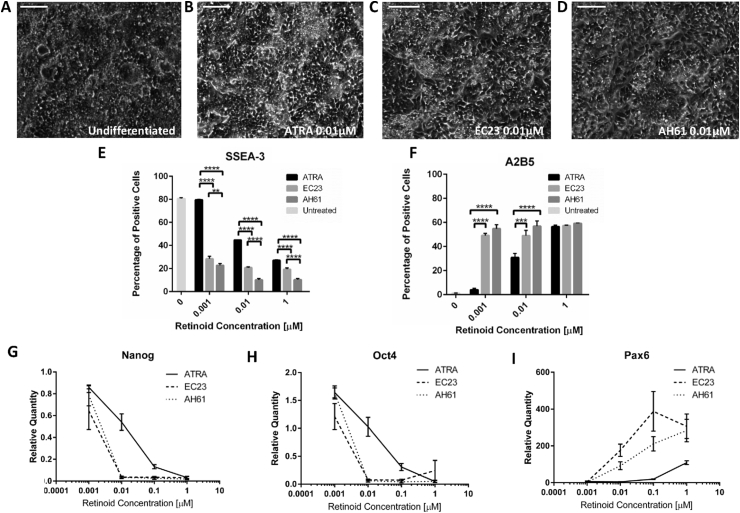
Induction of stem cell differentiation by retinoic acid. Representative phase contrast images of stem cells cultured as 2D monolayers (A) and subsequently treated with ATRA (B), EC23 (C) and AH61 (D) for 7 days. Flow cytometry (E) analysis of cellular expression of the stem cell marker, SSEA-3 (E) and the early neuronal marker, A2B5 (F) from 2D monolayers treated with retinoid compounds for 7 days (data represent mean ± SEM, n = 3). * = p < 0.05, ** = p < 0.01, *** = p < 0.001, **** = p < 0.0001. Analysis of gene expression of transcription factors; Nanog (G), Oct4 (H) and PAX6 (I) measured by Q-PCR. Expression is relative to the untreated control (data represent mean ± SD, n = 3). Scale bars: 200 μm.

Monolayers of TERA2.cl.SP12 stem cells were cultured with retinoid compounds for 7 days to induce neuronal differentiation. As can be seen in Fig. 1A, phase contrast micrographs show the typical stem cell morphology of undifferentiated cells, note cells are at high confluency and packed tightly into colonies, whereas monolayers of cells treated with 0.01 μM ATRA have areas where cells appear larger and lose their characteristic stem cell morphology (Fig. 1B). Similarly, monolayers treated with synthetic retinoids EC23 and AH61 (Fig. 1C and D) loose their stem cell-like morphology and possess a more differentiated phenotype.

Analysis of cell surface marker expression by flow cytometry indicated that the percentage of cells expressing the pluripotency marker SSEA-3 (Fig. 1E) declines with increasing concentration of all retinoid compounds tested. Treatment with the synthetic retinoids EC23 and AH61 results in significantly less SSEA-3 expression than treatment with ATRA at all concentrations. AH61 treatment induced SSEA-3 loss to a significantly greater extent than EC23 at all concentrations. A marker of early neuronal differentiation, A2B5 (Fig. 1F) is expressed highly upon retinoid treatment. Particularly at lower concentrations, 0.001 μM and 0.01 μM, A2B5 expression is significantly higher in cells treated with synthetic retinoids, compared to ATRA, indicating greater neural induction. However, as retinoid concentrations increase, the difference between the compounds becomes smaller and at 1 μM there is no significant difference in A2B5 expression among the compounds tested.

Expression of genes associated with pluripotency was also analysed through Q-PCR with *Nanog* and *Oct-4* exhibiting similar expression patterns following retinoid treatment (Fig. 1G and H). Both transcription factors were highly expressed at lower concentrations of all compounds and expression was lost in a dose-dependent manner as concentrations increased. EC23 and AH61 induced loss of expression of the transcription factors at much lower concentrations, than ATRA treatment, suggesting an enhanced potency of the synthetic molecules compared to naturally occurring ATRA. *Pax6,* a transcription factor involved in neural commitment, was expressed in a dose-dependent manner with increasing retinoid concentration. *Pax6* was expressed at much higher levels in cells treated with the synthetic molecules, EC23 and AH61, compared with ATRA (Fig. 1I). EC23 treatment induces maximal *Pax6* expression; suggesting EC23 promotes neural commitment and differentiation to a greater extent than the other molecules at the same concentration, further indicating that EC23 is a more potent inducer of neural differentiation.

### 3.2 Development and characterisation of a robust and reproducible pluripotent stem cell derived model of neurite outgrowth in 2D culture

**Fig. 2:**
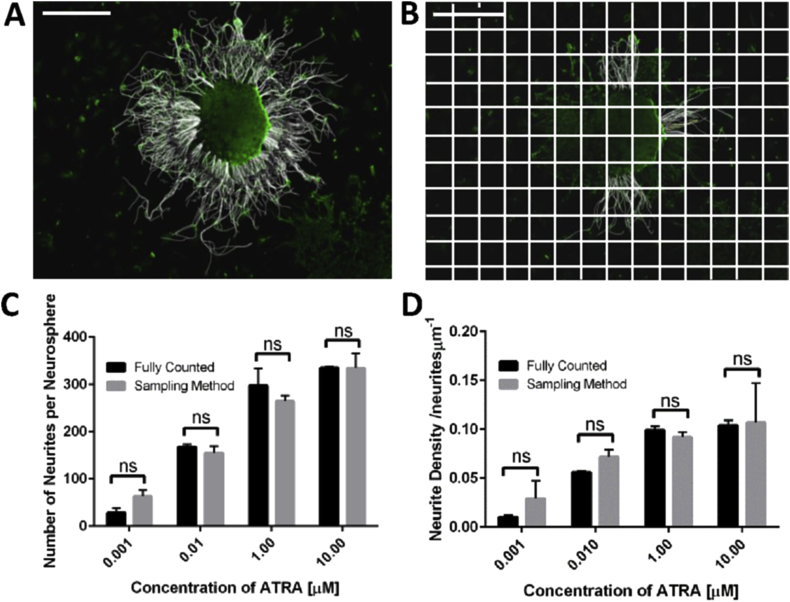
Development of an efficient method for the quantification of neurite outgrowth. Representative images showing TUJ-1 positive neurites (green) from neurospheres have been traced using image J software (white). All neurites from each neurosphere were counted (A) and compared with a sampling method (B) (see text for further details). Scale bars: 500 μm. Quantification of the number of neurites per neurosphere (C) and neurite density (D) for neurospheres differentiated with a range of concentrations of ATRA (0.001 μM-10 μM), and quantified using sampling method (data represent mean ± SEM, n = 2–8). No significant (ns) difference was found between counting all neurites (fully counted) and the more efficient sampling method developed. (For interpretation of the references to colour in this figure legend, the reader is referred to the web version of this article.)

A sampling procedure was developed to establish an efficient and accurate method to measure neurite outgrowth. Stem cell derived neurospheres, differentiated with increasing concentrations of ATRA, were first analysed by tracing every neurite per neurosphere using image analysis software (Fig. 2A). The same data set was then analysed again by overlaying a grid onto the image and randomly selecting three squares per image to quantify neurite outgrowth (Fig. 2B). Although neurite outgrowth may not be entirely consistent around each neurosphere, the two data sets were compared and a statistical analysis demonstrated no significant difference in the average number of neurites calculated per neurosphere (Fig. 2C) or the density of the neurites (Fig. 2D) when using either of the two methods of quantification. The sampling method was subsequently used as a satisfactory method of neurite quantification.

**Fig. 3:**
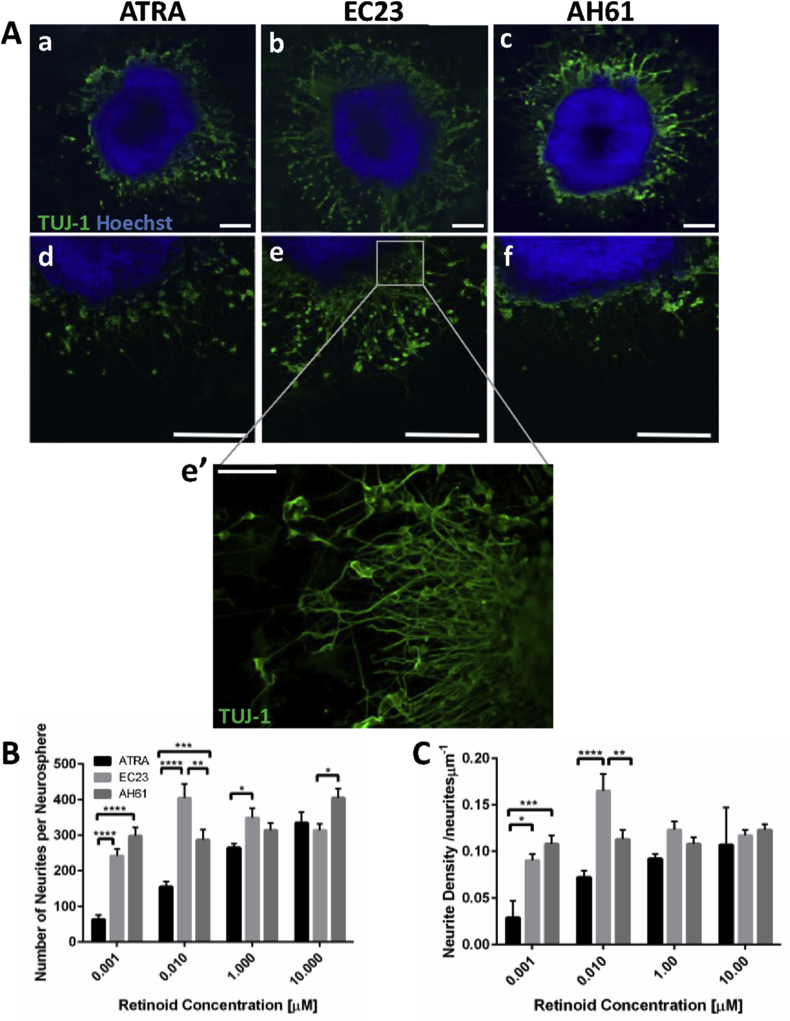
Induction of neural differentiation using natural and synthetic retinoids. Representative confocal images of neurospheres (A) differentiated with 0.01 μM ATRA (a,d), EC23 (b,e) and AH61 (c,f) and subsequently cultured in 2D. TUJ-1 positive neurites are highlighted in green, and nuclei in blue. Scale bars: 200 μm. High magnification image of neurites from a typical neurosphere differentiated with 0.01 μM EC23 (e’). Scale bar: 100 μm. Quantification examining the number of neurites per neurosphere (B) (data represent mean ± SEM, n = 15–24), and the number of neurites per μm of aggregate circumference, neurite density (C) (data represent mean ± SEM, n = 15–24). * = p < 0.05, ** = p < 0.01, *** = p < 0.001, **** = p < 0.0001. (For interpretation of the references to colour in this figure legend, the reader is referred to the web version of this article.)

The ability of synthetic retinoids to induce neurite outgrowth from neurospheres was analysed over a range of concentrations (0.001 μM–10 μM). Neurite outgrowth was observed using immunofluorescence (Fig. 3A), whereby nuclei (blue, Hoechst+) remained within the central cell aggregate while neurites (green, TUJ-1+) extended radially from the central aggregate body. Cell aggregates differentiated with ATRA (Fig. 3Aa,d) appeared to have produced less neurites and neurite outgrowth appears to be less uniform, than those aggregates differentiated with the same concentration of EC23 (Fig. 3Ab,e) or AH61 (Fig. 3Ac,f). Quantification revealed that at lower concentrations (0.001 μM and 0.01 μM), neurospheres differentiated with the synthetic retinoids produced significantly more neurites per cell aggregate (Fig. 3B). This was significant when normalised to aggregate size and expressed as neurite density (Fig. 3C) when compared to those differentiated with ATRA. However, at higher retinoid concentrations (1 μM and 10 μM), the differences in neurite outgrowth between the compounds became less pronounced, with the density of neurite outgrowth not significantly changing between the molecules tested. This is likely due to the maximal capacity of neurite outgrowth from the neurospheres being reached and increasing the concentration of retinoid molecules did not increase neurite outgrowth further. The optimum condition for inducing neuritogenesis from stem cell derived neurospheres was judged to be 0.01 μM EC23. This is because 0.01 μM EC23 induced significantly more neurites per neurosphere of greater density than aggregates differentiated with any of the other molecules tested. This optimal set of conditions was used to induce neuronal differentiation and significant neurite outgrowth for the remainder of this study.

**Fig. 4:**
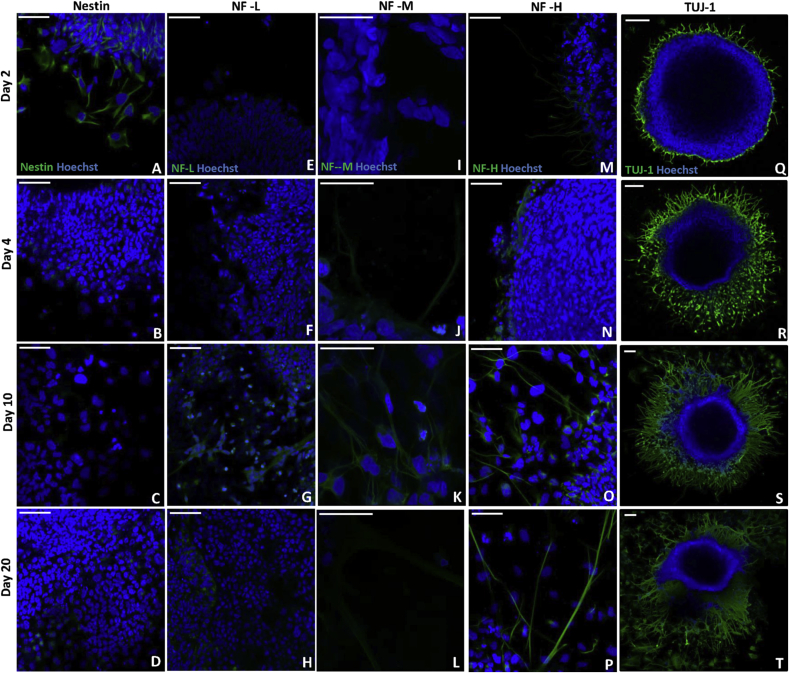
Characterisation of neuronal differentiation through the stages of neurite outgrowth from neurospheres over 20 days in 2D culture. Expression over time of nestin (A–D), neurofilament-L (E–H), neurofilament-M (I–L), neurofilament-H (M–P), and TUJ-1 (Q–T). Scale bars: (A–P): 50 μm, (Q–T): 100 μm.

To further characterise neuritogenesis by stem cell aggregates differentiated with 0.01 μM EC23, expression of known cytoskeletal markers were analysed over time. Nestin, a marker of neural stem cells, was expressed early in culture (Fig. 4A–D), mainly at day 2, whereas more intermediate markers of neurite outgrowth such as neurofilament-L (Fig. 4E–H) and neurofilament-M (Fig. 4I–L) are expressed later, during the intermediate stages (days 4–10) of the neurite outgrowth period. Neurofilament-H is a mature marker of neurite outgrowth and *in vivo* is expressed postnatally. Neurofilament-H was expressed predominantly from day 10, with greatest expression at day 20 in extended cultures (Fig. 4M–P). TUJ-1, a pan neuronal marker highlights the extensions of neurites from the aggregate over time (Fig. 4Q–T), with neurites beginning to extend at day 2 and increase in length over the 20 day culture period.

### 3.3 Development of a novel stem cell derived neurite outgrowth model within a 3D culture system

**Fig. 5:**
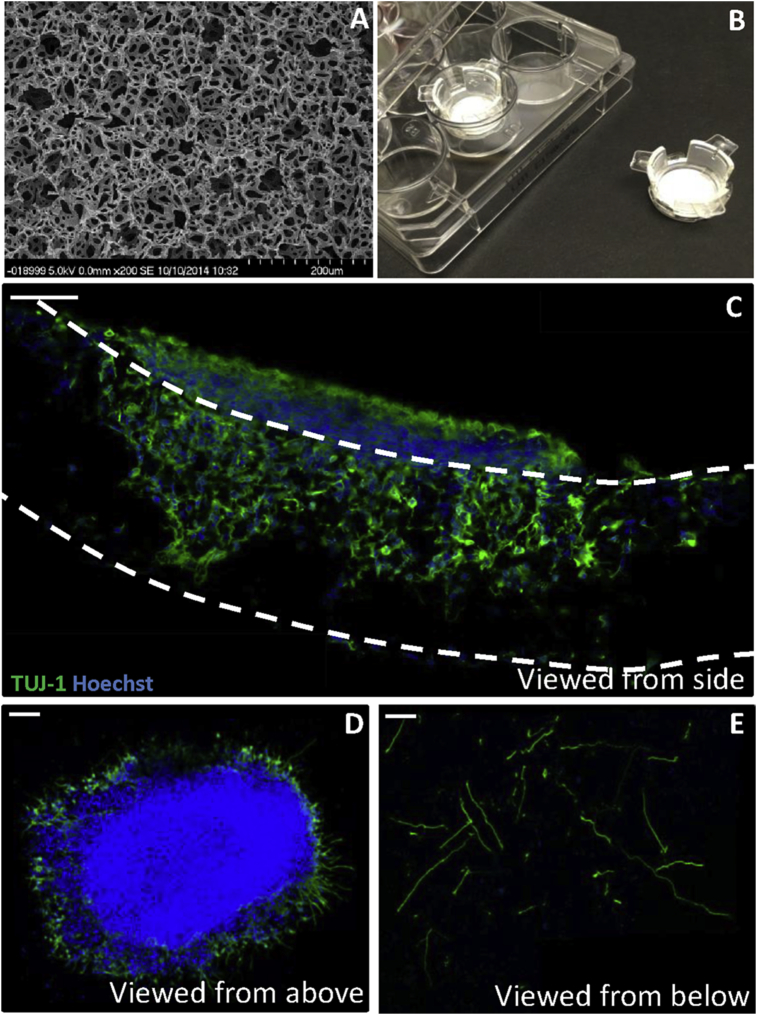
Development of a novel 3D model of neurite outgrowth. Scanning electron micrograph demonstrating the internal structure of the scaffold membrane (A) with voids of 40 μm and interconnecting windows of 13 μm. Photograph of Alvetex^®^ Scaffold 12-well inserts (B). Extensive neurite outgrowth can be observed within the 3D scaffold (C) with TUJ-1+ positive neurites staining green and nuclei blue, imaged using confocal microscopy. The majority of the neurosphere remains on top of the scaffold membrane (D) with neurites growing into the 3D material. Neurites can be visualized on the bottom of the scaffold (E), having grown through the 200 μm thick Alvetex^®^ Scaffold membrane. Scale bars: 100 μm. (For interpretation of the references to colour in this figure legend, the reader is referred to the web version of this article.)

Using the neurosphere model of neurite outgrowth developed herein, we then combined this system with established 3D cell culture technology. Building on previous work we have shown enhanced neuritogenesis on 3D Alvetex^®^ Scaffold membranes (Hayman et al., 2004, Hayman et al., 2005). Alvetex^®^ Scaffold is a highly porous scaffold membrane specifically developed for 3D cell culture applications (Fig. 5). The internal structure of Alvetex^®^ Scaffold is highlighted using scanning electron microscopy (SEM) (Fig. 5A) and comprises of 40 μm on average voids and 13 μm interconnecting windows. Membranes of Alvetex^®^ Scaffold are suspended in well inserts and provide an appropriate substrate on which to perform 3D cell culture (Fig. 5B).

Neurospheres differentiated with 0.01 μM EC23, were cultured on Alvetex^®^ Scaffold coated with poly-d-lysine and laminin, for 10 days in the presence of mitotic inhibitors. A cross-section of the Alvetex^®^ Scaffold reveals that the neurosphere perikarya predominantly remain on top of the scaffold membrane with few nuclei (blue, Hoechst+) penetrating into the material (approximately 5–10%). In contract, extensive neurite outgrowth (green, TUJ-1+) can be observed inside the scaffold membrane (Fig. 5C). The scaffold provides an environment and physical 3D space for neurites to grow and develop. A view from above shows the aggregate itself remaining on top of the membrane (Fig. 5D). Neurites penetrated the entire 200 μm thickness of the Alvetex^®^ Scaffold, as visualized on the bottom surface of the membrane (Fig. 5E). The number of neurites can subsequently be counted as a measure of neurite outgrowth through the 3D environment of the scaffold.

### 3.4 Application of novel 3D neurite outgrowth model to study neurite inhibition through co-culture with glioma cells

Using the neurosphere model of neurite outgrowth combined with 3D Alvetex^®^ Scaffold technology, we demonstrated the application of this system to study inhibition of neurite outgrowth. It is well known that neurite outgrowth is suppressed by inhibitory mechanisms in the glial scar after injury to the spinal cord (Chen et al., 2000, Prinja et al., 2000, Silver and Miller, 2004, McKeon et al., 1991). Our novel model of 3D neurite outgrowth provides an opportunity to co-culture and study growing neurites interacting with glial derivatives as a method to investigate these inhibitory processes.

**Fig. 6:**
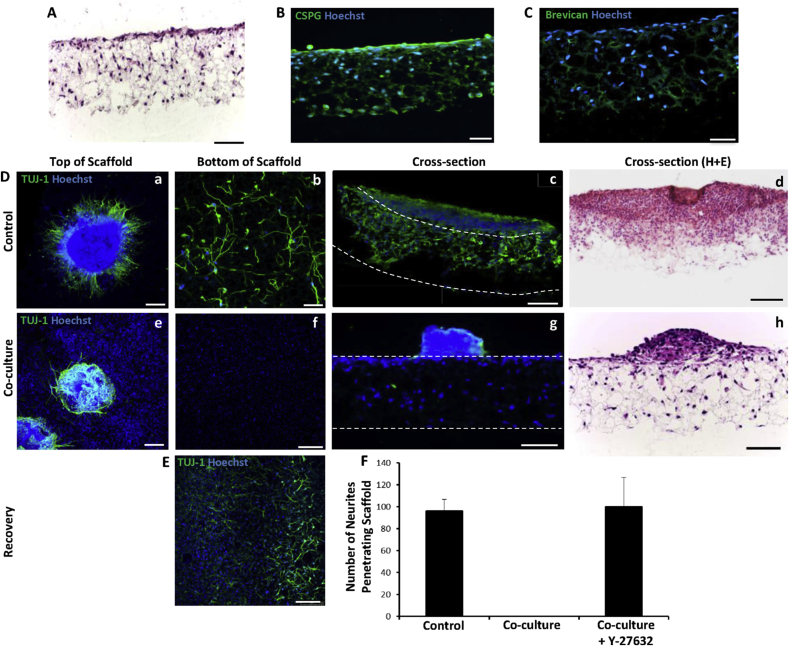
Development of a novel 3D co-culture model to study the interactions between neurons and glial cells. Representative heamatoxylin and eosin staining of U118MG cells cultured alone within a 3D scaffold (A) (scale bar: 100 μm), along with CSPG (B) and brevican (C) immunostaining (scale bars: 50 μm). In the absence of co-cultured glioma cells, neurites sprout from the neurospheres and growth can be observed through the scaffold and visualized from the bottom of the membrane (D a-c). During co-culture, the glioma cells inhibit neurite outgrowth. Neurites grow over the surface of the neurosphere (D e) and do not penetrate the scaffold in the presence of the glioma cells (D f-h). Cross-sections of both control and co-cultured neurospheres were stained both with the neuronal marker TUJ-1+ (D c,g) and with haematoxylin and eosin staining (D d,h). Glioma cell induced neurite inhibition can be suppressed by application of the ROCK inhibitor, Y-27632 and significant numbers of neurites penetrate the scaffold as visualized from the bottom of the scaffold (E). Quantification of neurite outgrowth from neurospheres cultured with and without glioma cells and with the addition of Y-27632 (F) (data represent mean ± SEM, n = 3). Scale bars: (D a,b,e,f, E): 250 μm, (D c,g,d,h): 100 μm.

To simulate some of the interactions between cell types involved in the glial scar that arise post-spinal cord injury, we co-cultured neurospheres with the astroglioma cell line, U-118 MG. This was made possible due to the 3D nature of Alvetex^®^ Scaffold, as both cell types could be brought together within a 3D environment. To establish the 3D culture of U-118 MG cells, cells were cultured with mitotic inhibitors for 10 days in the scaffold membrane (Fig. 6A). In 3D culture, the glioma cells positively expressed chondroitin sulphate proteoglycans (CSPGs), as revealed by anti-CSPG staining (Fig. 6B), and more specifically the CSPG brevican (Fig. 6C). These molecules are known to inhibit neurite outgrowth and are thought to play a major role in supressing neural regeneration in the damaged spinal cord (Clemens et al., 2013).

In the absence of glioma cells, neurospheres can be observed on top of the scaffold (Fig. 6D,a; control) with extensive neurite outgrowth penetrating the depth of the scaffold (Fig. 6D,b). Extensive neurite outgrowth can be observed within the scaffold through both TUJ-1 positive extensions (Fig. 6D,c) and cytoplasmic extensions observed through haematoxylin and eosin staining (Fig. 6D,d). The nuclei of the glioma cells (blue, Hoechst+) can be observed throughout the scaffold (Fig 6D,e-g). In the presence of U-118 MG cells, few neurites radiated from the neurosphere but instead grew over the surface of the cell aggregate (Fig. 6D,e) and there was no evidence of any neurites having penetrated the scaffold (Fig. 6D,f-h). The addition of the ROCK inhibitor, Y-27632, to the co-culture system promoted neurite outgrowth, overcoming glioma cell mediated neurite inhibition. A view from the bottom of the scaffold allowed visualisation of neurites penetrating the scaffold, even in the presence of the glioma cells (Fig. 6E). Quantification of the numbers of neurites observed on the bottom of the membrane showed that treatment by Y-27632 overcame neurite inhibition and enabled the recovery of neurite outgrowth to the same levels as observed in the control condition (Fig. 6F). As significant neurite outgrowth is observed in the presence of glioma cells and ROCK inhibition, it suggests that the inhibition of neurite outgrowth observed is not due to physical blockage of neurite growth by the glioma cells in the Alvetex^®^ scaffold, but rather is due to overcoming inhibitory factors expressed by them.

## 4 Discussion

Neuritogenesis is an essential process that enables the formation of complex neuronal signalling pathways. The inability of neurons to undergo neurite outgrowth is common to many nervous system disorders. Impaired neurite outgrowth has been implicated in pathologies including central nervous system trauma (Chen et al., 2000, Prinja et al., 2000, Silver and Miller, 2004, McKeon et al., 1991), Down's Syndrome (Bahn et al., 2002, Murtomaki et al., 1992), schizophrenia (Ozwki et al., 2003) and neurodegenerative diseases such as Alzheimer's (Petratos et al., 2008, Postuma et al., 2000) and Parkinson's Disease (Takenouchi et al., 2001). As neurite inhibition may be common to such a large variety of neurological disorders, it highlights the need for a well characterised and physiologically representative model of the process that can screen the ability of small molecules to recover such inhibition and provide potential therapeutic strategies.

Current neurite outgrowth models routinely used within neuroscience research generally utilise the differentiation of cell monolayer cultures, whether it be of stem cells (Tegengem et al., 2011, Satoh et al., 1997, Pewsey et al., 2010, Roloff et al., 2015), cancer cells (Ferrari-Toninelli et al., 2004, Tucholski et al., 2001, Ross et al., 1983) or primary animal derived cells (Clagett-Dame et al., 2006, Balgude et al., 2001, Fitzgerald et al., 1993). However, quantification of neurite outgrowth in populations of cells grown as monolayers can be challenging using image analysis software, as it can be difficult to individually trace each neurite back to its parent cell body within an intricate neurite network (Radio and Mundy, 2008). The methodology described in this study utilises a spheroid culture technique that promotes human neurite outgrowth radially from a single central cell aggregate. We show that neurites can be easily traced, using an efficient sampling method to enhance quantification. This is more amenable to scaling up and can more easily be adapted for high throughput screening.

The use of synthetic retinoids to differentiate pluripotent stem cells has been evaluated within this study. ATRA is a naturally occurring metabolite of vitamin A is often used to promote neuronal differentiation *in vitro* (Bain et al., 1996, Jones-Villeneuve et al., 1982, Jones-Villeneuve et al., 1983, Andrews, 1984). However ATRA is unstable, and breaks down when exposed to light and heat which is why its use *in vitro* is limited and can give rise to inconsistency and variation (Gurwitz and Cunnigham, 1988). We have shown the ability of synthetic, stable retinoids to induce neural differentiation is significantly enhanced compared with ATRA (Clemens et al., 2013, Christie et al., 2008). Synthetic retinoid EC23 was found to be a potent inducer of neurite outgrowth to a much greater extent than the other molecules tested. This was noted especially at lower concentrations where EC23 had a greater effect than ATRA on the reduction of stem cell markers and increase in neuronal markers. This is most likely due to its increased stability and potency as unlike ATRA, EC23 contains a non-isomerisable conjugated linker unit and does not readily photoisomerise (Clemens et al., 2013, Christie et al., 2008).

*In vitro* models of neurite outgrowth generally utilise conventional 2D cell culture methods. However, this has limitations, as the growth of cells in 2D culture is far removed from the complex environment that cells experience *in vivo* (Knight and Przyborski, 2015). Cells cultured on 2D substrates flatten and remodel their cytoskeleton resulting in changes that do not resemble the function of their *in vivo* counterparts (Knight and Przyborski, 2015). Previously 3D cell culture technology has been found to enhance neuritogenesis in a stem cell derived model (Hayman et al., 2004, Hayman et al., 2005). In this study we have combined the consistency of synthetic retinoids with a robust method of producing neurospheres and quantified neurite outgrowth in 2D and 3D culture. Culturing neurospheres in a 3D environment results in neurites growing in a more physiological geometry without the limitations of 2D culture. This is particularly important when screening the ability of small molecules to promote neurite outgrowth and overcome neurite inhibition, as a more physiologically relevant model could improve the accuracy of predictions as to whether molecules may be suitable as potential therapeutics (Knight and Przyborski, 2015).

In addition, the model described in this study has the advantage of being derived from human cells, and is more relevant for the study of human diseases. Characterisation of this model and assessment of markers associated with neural differentiation is consistent with previously published evidence (Przyborski, 2001; Przyborski et al., 2000, Przyborski et al., 2003, Przyborski et al., 2004; Stewart et al., 2004; Coyne et al., 2011), suggesting that the neurite outgrowth observed shares similarities with the early stages of nervous system development.

We have described a potential application of this technology to support investigation into the molecular mechanisms that underpin neurite inhibition within the injured spinal cord. An inhibitory environment arises post-spinal cord injury, known as the glial scar. One important component of the glial scar are reactive astrocytes that upon injury become reactive and secrete CSPG inhibitory molecules (Fawcett and Asher, 1999, Yiu and He, 2006b, Seidenbecher et al., 2002, Dou and Levine, 1994, Xu et al., 2015). Immunofluorescence of U-118 MG cultured within the 3D scaffold highlights expression of such CSPG molecules, with specific staining for the molecule brevican, a member of the lectican family (Yiu and He, 2006b), indicating that the glioma cell line expresses CSPGs when cultured within the 3D environment.

Neurospheres derived using the methods described herein were co-cultured with U-118 MG glioma cells in a 3D culture environment in an attempt to simulate aspects of the cellular interactions between neurites and astrocytes of the glial scar. In the presence of the U-118 MG cells, neurite outgrowth was completely lost. As there is evidence that U-118 MG cells express CSPGs, this may explain the inhibition of neurite outgrowth observed. We propose that our model provides a novel 3D system to study the interactions between neuronal and glial populations and also to investigate molecular mechanisms such as suppression of neurite growth by chemical inhibition such as CSPGs. Small molecules can then be applied to the system to investigate their ability to overcome such inhibition, an example of such a molecule is Y-27632 the ROCK inhibitor that has previously been established to overcome CSPG mediated inhibition (Lingor et al., 2007). Here we demonstrate the ability of Y-27632 to overcome CSPG mediated neurite inhibition within the context of our 3D co-culture model, whereby glioma cells are in contact with developing neurites. The ability of compounds to overcome such inhibition is important in the development of potential therapeutic strategies and the discovery of new pharmaceuticals.

In conclusion the present study describes the optimisation of a robust, reproducible and novel 3D model of neurite outgrowth that can be quantified easily and accurately to assay neurite outgrowth. There are many potential applications for such a model of neuritogenesis including, screening potential therapeutics, studying the developmental process of neuritogenesis or investigating the molecular mechanisms that underpin neurite inhibition within many neurological disorders. The systems we have developed herein also utilise efficient methods to measure and quantify neurites in 2D and 3D culture. This is essential for the use of such a model as a screening tool to select potential molecules that may be able to overcome neurite inhibition for therapeutic use.
